# Learning from autopsies

**DOI:** 10.3402/gha.v7.24053

**Published:** 2014-03-11

**Authors:** Kieran Walsh

**Affiliations:** BMJ Learning, BMA House, London, UK

Dear Editor,

Tette et al. present a fascinating insight into the utility of autopsies from the point of view of clinicians at their institution (1). Importantly, the majority of respondents felt that autopsies were useful for the purpose of medical education. However, despite this, only a minority of clinicians attended autopsy sessions and there was clearly limited interaction between clinicians and pathologists.

Could medical education be a method of improving this situation? It is likely that it could – but it would more likely be successful if medical education were implemented in a planned and programmatic way. There are a number of means by which this could happen. First, attendance at autopsies could be a required part of the curriculum at undergraduate and postgraduate levels. Learners at these levels are often curriculum-driven, and so changing the curriculum is likely to be an effective means of changing their behaviour. Second, fully-qualified doctors should be able to claim personal continuing professional development credits for attendance at autopsies, and equally pathologists should be able to claim credits for attending educational clinicopathological case conferences. Learners at this level often need to accrue credits, so once again this tactic is likely to be effective for them. Third, the results of autopsies could become not just part of audit, but part of continuous clinical quality improvement systems in hospitals. The core purpose of autopsies is to improve care, so it makes sense to join them up with formal mechanisms of quality improvement. Fourth, feedback from the autopsy could be distributed more widely within the team and also within a best practice format. Doctors often fear receiving feedback – they worry that such feedback might be negative or might be presented in a personal and damaging way. However, feedback from autopsies need not be like this. Such feedback can be specific and result in clear plans that are achievable, measurable, and realistic. Finally, autopsies could be integrated into programmes of assessment. Undergraduates might be assessed on their attendance at and learning from autopsies; at the postgraduate level, the results of autopsies might become part of work-based assessments. It is an old adage that ‘assessment drives learning’, and making autopsies part of assessments will likely drive learning in this field (2).

Doctors at all levels have much to learn from autopsies, and using these methods is likely to be an effective way of leveraging the benefits of these unique teaching tools.

Yours Sincerely, *Kieran Walsh*
BMJ Learning, BMA House, London, UK

## References

[1] Tette E, Yawson AE, Tettey Y (2014). Clinical utility and impact of autopsies on clinical practice among doctors in a large teaching hospital in Ghana. Glob Health Action.

[2] Miller G (1990). The assessment of clinical skills/competence/performance. Acad Med.

